# Intrapartum Fetal Compromise in Late-Onset Fetal Growth Restriction Using the Modified Myocardial Performance Index: A Prospective Cohort Study

**DOI:** 10.3390/medicina62030572

**Published:** 2026-03-19

**Authors:** Yücel Kaya, Verda Alpay, Emrah Dagdeviren, İlteriş Yaman

**Affiliations:** 1 Division of Perinatology, Department of Obstetrics and Gynecology, Basaksehir Cam and Sakura City Hospital, Istanbul 34480, Turkey; verda_alpay@yahoo.com; 2 Department of Obstetrics and Gynecology, Basaksehir Cam and Sakura City Hospital, Istanbul 34480, Turkey; dagdeviren_emrah_58@hotmail.com; 3 Division of Gynecologic Oncology Surgery, Department of Obstetrics and Gynecology, Basaksehir Cam and Sakura City Hospital, Istanbul 34480, Turkey; drilterisyaman@gmail.com

**Keywords:** fetal growth retardation, fetal distress, echocardiography, parturition

## Abstract

*Background and Objectives*: Predictive performance of the modified myocardial performance index (Mod-MPI) for emergency cesarean delivery secondary to intrapartum fetal compromise (IFC) was examined in late-onset fetal growth restriction (FGR) or small-for-gestational-age (SGA) pregnancies. *Materials and Methods*: This prospective observational cohort comprised 120 singleton term pregnancies affected by late-onset FGR or SGA, classified in line with the Delphi consensus criteria, for whom a trial of vaginal delivery was planned. The primary endpoint was emergency cesarean delivery indicated by IFC, while the secondary endpoint was the development of composite adverse perinatal outcomes (CAPO). Measurements of Mod-MPI, the umbilical artery, the middle cerebral artery, and the cerebroplacental ratio (CPR) were performed within the final 72 h before delivery and were blinded to the clinicians managing the intrapartum period. *Results*: IFC constituted 28.3% (*n* = 34) of the study cohort. The IFC group exhibited significantly higher Mod-MPI values and lower CPR values (*p* < 0.001). In multivariable analysis, elevated Mod-MPI (≥0.61) and reduced CPR (<5th percentile) were identified as independent predictors of IFC. On receiver operating characteristic analysis, Mod-MPI demonstrated superior discriminative performance compared with CPR for predicting IFC (area under the curve [AUC]: 0.835 vs. 0.759). In contrast, CPR showed the highest diagnostic performance for predicting CAPO (AUC: 0.779). *Conclusions*: Mod-MPI reflects subclinical cardiac dysfunction in fetuses with late-onset FGR and SGA and represents a valuable parameter for predicting tolerance to acute intrapartum stress. Rather than routine implementation, it appears most appropriate as a complementary tool contributing to intrapartum risk assessment in selected high-risk cases.

## 1. Introduction

The presence of fetal growth restriction (FGR) or a small-for-gestational-age (SGA) phenotype is linked to substantially higher rates of perinatal morbidity and mortality [1,2]. Fetuses in this group have a markedly increased risk of intrapartum fetal compromise (IFC), neonatal intensive care unit (NICU) admission, and perinatal mortality [3,4,5]. A central challenge in clinical management is the accurate distinction between constitutionally small but physiologically healthy SGA fetuses and truly pathological FGR resulting from uteroplacental insufficiency [6]. As defined by the international Delphi consensus, fetal growth restriction (FGR) encompasses fetuses with more pronounced biometric impairment and/or associated Doppler abnormalities, whereas small-for-gestational-age (SGA) refers to fetuses with milder biometric smallness and normal Doppler findings [7]. Decisions regarding delivery timing and mode are critical to balance minimizing intrauterine hypoxia against avoiding iatrogenic prematurity. Nevertheless, current clinical assessment approaches frequently remain inadequate for reliably identifying which fetuses will tolerate vaginal delivery and which are likely to decompensate during the intrapartum period.

Standard approaches to the assessment of fetal well-being include Doppler velocimetry of vascular structures—specifically the umbilical artery (UA), middle cerebral artery (MCA), and ductus venosus (DV)—complementing cardiotocography (CTG) and the biophysical profile [8,9]. In cases of late-onset FGR, isolated UA and MCA Doppler indices may be insufficient to identify evolving fetal hypoxia, whereas the cerebroplacental ratio (CPR), which more sensitively reflects the brain-sparing effect, has gained increasing attention [10]. Nevertheless, the contemporary literature indicates that both CPR and conventional Doppler parameters have limited accuracy in predicting the development of intrapartum fetal distress and the subsequent need for emergency cesarean delivery [11,12]. Accordingly, particularly in pregnancies in which a trial of vaginal delivery is planned, these limitations highlight the clinical need for novel parameters that more directly reflect intrapartum adaptive capacity.

The myocardial performance index, also referred to as the Tei index, integrates isovolumetric contraction time (ICT), isovolumetric relaxation time (IRT), and ejection time (ET) to assess global ventricular function [13]. Hernandez-Andrade et al. introduced the modified myocardial performance index (Mod-MPI), improving measurement reproducibility and enabling a more reliable assessment of fetal cardiac function [14]. Nevertheless, it remains unclear whether antenatal Mod-MPI can reliably distinguish fetuses capable of withstanding intrapartum stress from those at increased risk.

The primary objective of this study was to determine the predictive value of antenatal Mod-MPI measurements for cesarean delivery due to intrapartum fetal compromise (IFC) in fetuses diagnosed with FGR and SGA. A secondary aim was to explore the relationship between this parameter and composite adverse perinatal outcomes (CAPO). We hypothesized that elevated antenatal Mod-MPI values reflect limited cardiac reserve, thereby reducing the ability to adapt to labor and increasing the risk of IFC-related cesarean delivery.

## 2. Materials and Methods

### 2.1. Study Design and Setting

This study was designed as a prospective observational cohort study and was conducted between 2024 and 2025 at the Department of Perinatology of Başakşehir Çam and Sakura City Hospital, a tertiary referral center. The study involved no randomization or interventions, relying on antenatal sonographic data from routine clinical follow-up. Participants were followed throughout the perinatal period, and all obstetric management was performed in accordance with established institutional protocols. This research followed the Strengthening the Reporting of Observational Studies in Epidemiology (STROBE) statement for reporting observational data [15]. The research protocol complied fully with the ethical principles of the Declaration of Helsinki and was approved by the local ethics committee (Approval No. KAEK-11/16.10.2024.188). All participants provided written informed consent once they were fully apprised of the study’s aims.

### 2.2. Participants

The study population consisted of consecutive pregnancies in which late-onset (≥32 weeks of gestation) FGR or SGA was identified during antenatal follow-up. Late-onset FGR was defined according to the Delphi consensus criteria [16]. Accordingly, late-onset FGR classification relied on an abdominal circumference (AC) or estimated fetal weight (EFW) < 3rd percentile, or meeting a minimum of two conditions among: (1) AC or EFW below the 10th percentile; (2) AC or EFW crossing more than two quartiles on growth centiles; and (3) Doppler pathology indicated by CPR < 5th percentile or umbilical artery pulsatility index (UA PI) > 95th percentile. Fetuses with AC or EFW measurements between the 3rd and 10th percentiles in the absence of growth deceleration or Doppler abnormalities were classified as SGA.

The inclusion criteria were defined as confirmation of gestational age by reliable first-trimester ultrasonography (crown–rump length [CRL]), AC or EFW < 10th percentile, delivery at ≥37 weeks of gestation, and the absence of absolute obstetric contraindications to a trial of vaginal delivery. Exclusion criteria were multiple pregnancies, structural or chromosomal fetal anomalies, non-vertex presentation, prior uterine surgery, oligohydramnios, severe Doppler abnormalities (absent/reversed end-diastolic flow in the UA, or absent/reversed a-wave in the DV), active labor at admission (regular uterine contractions with cervical dilatation ≥ 4 cm or a Bishop score > 6), and suboptimal Doppler or Mod-MPI measurements. In addition, cases in which informed consent was not provided or delivery occurred at another institution were excluded from the analysis.

### 2.3. Variables

We defined the primary endpoint as emergency cesarean delivery necessitated by IFC. The secondary outcome was the occurrence of CAPO, defined by the presence of at least one of the following criteria: (i) a 5 min Apgar score < 7, (ii) admission to NICU, or (iii) perinatal mortality.

The independent variables of the study included maternal demographic characteristics (maternal age, body mass index [BMI], smoking status), maternal comorbidities (chronic hypertension, pregestational diabetes, thyroid disease), pregnancy-specific complications (gestational diabetes, pregnancy-induced hypertension, intrahepatic cholestasis), clinical diagnostic variables (FGR, SGA), obstetric characteristics (gravida, parity, gestational age at delivery), and antenatal ultrasonographic parameters. Ultrasonographic evaluation included fetal biometry (AC, EFW), UA PI, middle cerebral artery pulsatility index (MCA PI), ductus venosus pulsatility index (DV PI), and CPR. In the assessment of fetal cardiac function, peak E and A wave velocities at the level of the mitral valve, ICT, IRT, ET, and Mod-MPI were recorded.

### 2.4. Data Sources/Measurement

Data were collected via prospective routine antenatal ultrasound measurements, with intrapartum and perinatal outcomes retrospectively verified from electronic medical records. All ultrasonographic examinations were performed within the 72 h preceding delivery using an ARIETTA 850 ultrasound system (Hitachi Medical Corporation, Tokyo, Japan) equipped with a 3.5 MHz convex transducer.

Fetal biometry, including biparietal diameter, head circumference, femur length, AC, and EFW, was performed using standard techniques in accordance with International Society of Ultrasound in Obstetrics and Gynecology (ISUOG) guidelines [17]. Percentile values for AC and EFW were calculated using the Hadlock reference charts [18,19].

Doppler assessment of the UA, MCA, and DV followed ISUOG guidelines using pulsed-wave velocimetry [20]. During periods without fetal breathing or movements, wall motion filter and pulse repetition frequency (PRF) settings were optimized to obtain three consecutive and symmetric waveforms. The ALARA (As Low As Reasonably Achievable) principle was applied throughout the examinations, with the thermal index maintained at ≤1.0. UA Doppler was sampled from a free-floating cord loop; MCA Doppler was sampled from the proximal third of the vessel near the circle of Willis, with the insonation angle kept near 0°. CPR was calculated as the ratio of MCA PI to UA PI. UA and CPR measurements were converted to percentile values using the reference charts of the Fetal Medicine Foundation (FMF) [21]. DV flow waveforms were obtained as a triphasic pattern in the midsagittal plane of the fetal abdomen from a vessel demonstrating aliasing on color Doppler, using a small sample volume and low wall filter settings, with alignment achieved without the need for angle correction.

Fetal left ventricular Mod-MPI was measured according to Hernandez-Andrade et al. [14]. A transverse plane of the fetal chest was utilized to capture an apical four-chamber view, followed by tilting the transducer to visualize the left ventricular outflow tract and setting the pulsed-wave Doppler sample volume to 3 mm. The Doppler sampling volume was adjusted to simultaneously encompass flow signals from the ascending aortic wall and the mitral valve leaflet, enabling concurrent recording of ventricular inflow and outflow waveforms. Measurements were obtained during periods without fetal movement or breathing, with the wall filter adjusted to 300 Hz, the Doppler sweep speed set to 15 cm/s, and the angle of insonation maintained at <20°. Using uniform waveforms derived from three consecutive cardiac cycles with a fetal heart rate within the physiological range, the interval between mitral valve closure and aortic valve opening was recorded as ICT, the interval between aortic valve closure and mitral valve opening as IRT, and the interval between aortic valve opening and closure as ET. The Mod-MPI was derived via the expression (ICT + IRT)/ET, based on the arithmetic mean of these measurements. In addition, for the assessment of diastolic function, peak velocities of the E wave, representing early diastolic mitral inflow, and the A wave, reflecting late diastolic atrial contraction, were measured, and the E/A ratio was recorded.

The study cohort was stratified into two main groups based on intrapartum CTG findings and mode of delivery. The IFC group consisted of cases in which emergency cesarean delivery was performed due to pathological fetal heart rate patterns classified according to the criteria of the International Federation of Gynaecology and Obstetrics (FIGO), including recurrent late decelerations, persistent bradycardia, or a sinusoidal pattern [22]. The non-IFC group included cases without pathological CTG findings during intrapartum surveillance who delivered vaginally (spontaneous or operative) or by cesarean delivery for obstetric indications other than IFC.

### 2.5. Bias

To minimize observer and performance bias, the obstetric team managing labor was blinded to antenatal Doppler and cardiac measurements. Similarly, the pregnant women enrolled in the study were unaware of the results of the ultrasonographic parameters. This approach ensured that all intrapartum decisions were based solely on standard obstetric indications and fetal heart rate patterns evaluated according to FIGO criteria as part of routine clinical protocols.

To reduce selection bias, all consecutive cases presenting during the study period and meeting the inclusion criteria were enrolled. To limit measurement bias, ultrasonographic assessments were conducted by a single experienced sonographer (YK).

Finally, a two-step strategy was implemented to address confounding bias. During the design phase, cases with clinical conditions—such as fetal arrhythmias, structural or chromosomal fetal anomalies, or oligohydramnios—that could independently influence intrapartum fetal heart rate patterns and decisions regarding mode of delivery, irrespective of antenatal ultrasonographic findings, were excluded from the study population. During the analysis phase, given the well-established influence of birth weight on fetal well-being and intrapartum clinical outcomes, statistical adjustment was performed in multivariable models to isolate the independent effects of Mod-MPI and Doppler parameters.

### 2.6. Statistical Analysis

SPSS software (version 26.0; IBM Corp., Armonk, NY, USA) was employed for the execution of all data analyses. Distributional properties of continuous variables were examined with the Kolmogorov–Smirnov test. Variables following a normal distribution were summarized using mean ± standard deviation, whereas those with non-normal distributions were reported as median (minimum–maximum). Categorical data were expressed as frequencies and percentages (%). Depending on data distribution, continuous variables were analyzed via Student’s *t*-test or Mann–Whitney *U* test. For categorical data, the Pearson chi-square test was employed, with Fisher’s exact test applied when expected frequencies were low. Receiver operating characteristic (ROC) curves were generated to assess the diagnostic performance of UA PI, MCA PI, CPR, and Mod-MPI for predicting IFC and CAPO, presenting the area under the curve (AUC) alongside 95% confidence intervals (CI). Optimal thresholds were derived using the Youden index, and the corresponding sensitivity and specificity metrics were subsequently obtained. To identify independent predictors of IFC, univariate logistic regression analysis was initially performed, and variables with *p* < 0.10, together with clinically relevant variables, were entered into multivariable logistic regression models. In multivariable analyses, birth weight was included as an adjustment variable because of its potential confounding effect. Results were reported as adjusted odds ratios (OR) with 95% CI. Model fit was evaluated using the Hosmer–Lemeshow goodness-of-fit test. Two-tailed statistical testing was applied, with *p* < 0.05 indicating statistical significance.

An a priori sample size calculation was performed using G*Power version 3.1 (Heinrich Heine University, Düsseldorf, Germany)**, ** based on the Mod-MPI parameter reported by Simsek and Kose [2,23]. Assuming an effect size of 0.714, a type I error probability (α) of 0.05, and a power (1 − β) of 0.80, the minimum required total sample size was calculated as 66 participants (n1 = 28; n2 = 38). During the study period, the IFC and Non-IFC groups included 34 and 86 patients, respectively, indicating that an adequate sample size was achieved. A post hoc power analysis was also performed using the same software to assess the statistical power of the between-group comparison for the primary variable, Mod-MPI. Based on an observed effect size of 1.36 between the two groups and a predefined type I error threshold (α = 0.05), the achieved statistical power (1 − β) was greater than 0.99, confirming that the study was sufficiently powered.

## 3. Results

**Figure 1:**
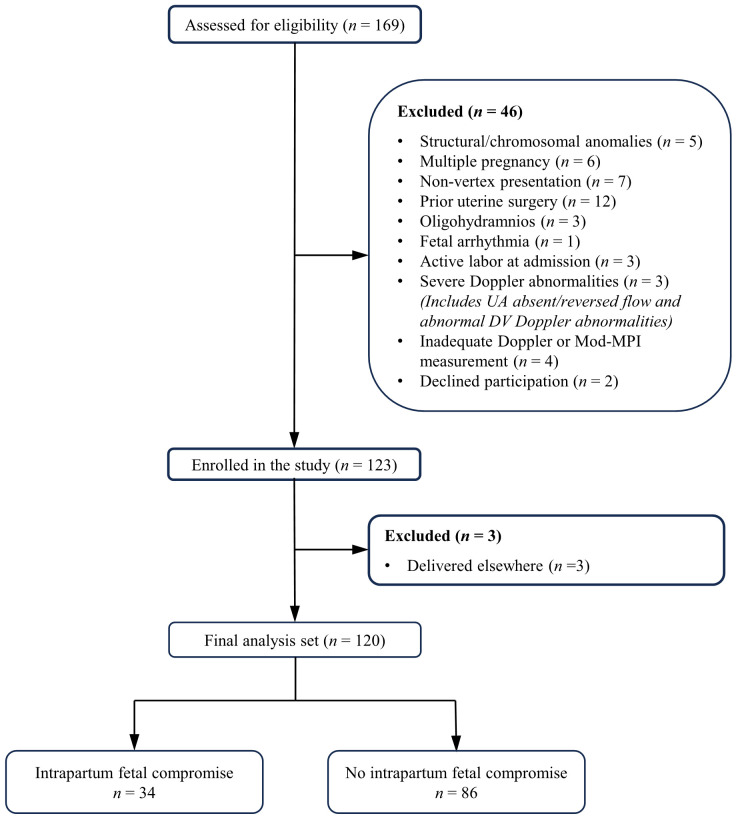
Flow diagram of patient enrollment, exclusion, and final study population. Abbreviations: UA, umbilical artery; DV, ductus venosus; Mod-MPI, modified myocardial performance index.

Of 169 cases screened, 46 were ineligible and 3 were excluded due to external delivery, yielding a final cohort of 120. Among these, 34 cases (28.3%) constituted the IFC group, whereas 86 cases (71.7%) formed the non-IFC group (Figure 1).

Table 1 summarizes the baseline demographic and clinical characteristics of the study groups. Maternal age, BMI, smoking status, obstetric history, comorbidities, and pregnancy-related complications did not differ significantly between groups. The proportion of cases diagnosed with FGR was significantly higher in the IFC group than in the SGA group (85.3% vs. 14.7%, *p* < 0.001). Gestational age at ultrasound was significantly lower in the IFC group (*p* < 0.001). In addition, the proportions of AC < 3rd percentile and EFW < 3rd percentile were significantly higher in the IFC group (*p* < 0.001 and *p* = 0.003, respectively). Regarding perinatal outcomes, the IFC group exhibited significantly reduced gestational age at delivery and birth weight (all *p* < 0.001). Furthermore, the incidence of NICU admission and CAPO was substantially elevated in the IFC group (all *p* < 0.001). The proportion of neonates with a 5 min Apgar score < 7 was similar between the groups, and no cases of perinatal mortality were observed in either group.

Table 2 compares antenatal fetal Doppler and cardiac function parameters. The IFC group exhibited significantly higher UA PI and a greater frequency of UA PI > 95th percentile, with lower MCA PI and CPR, and a higher prevalence of CPR < 5th percentile (all *p* ≤ 0.001). No significant difference was observed between the groups with respect to DV PI. When echocardiographic parameters of fetal cardiac function were evaluated, peak E and A wave velocities at the mitral valve level and the E/A ratio did not differ significantly between the groups. By contrast, ICT and IRT showed significant prolongation among cases with IFC relative to those without IFC, while ET was significantly shorter (*p* < 0.001, *p* = 0.004, and *p* < 0.001, respectively). Mod-MPI values were also significantly higher in the IFC group (*p* < 0.001).

**Figure 2:**
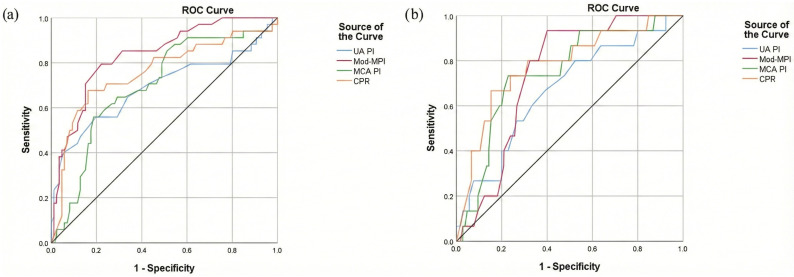
Receiver operating characteristic (ROC) curves comparing the predictive performance of Doppler parameters for (**a**) intrapartum fetal compromise and (**b**) composite adverse perinatal outcome. Curves are shown for umbilical artery pulsatility index (UA PI), middle cerebral artery pulsatility index (MCA PI), cerebroplacental ratio (CPR), and modified myocardial performance index (Mod-MPI). The diagonal black line represents the line of no discrimination.

Using ROC analysis, the diagnostic capability of ultrasonographic parameters in predicting IFC and CAPO was evaluated (Figure 2; Table 3). Regarding IFC prediction, Mod-MPI provided the greatest discriminatory ability (AUC = 0.835, 95% CI: 0.756–0.914; cutoff = 0.61; sensitivity = 79.4%; specificity = 77.9%; *p* < 0.001), followed by CPR (AUC = 0.759; *p* < 0.001), MCA PI (AUC = 0.699; *p* = 0.001), and UA PI (AUC = 0.691; *p* = 0.001). In contrast, CPR yielded the most robust discriminative ability for CAPO (AUC = 0.779, 95% CI: 0.649–0.908; cutoff = 1.24; sensitivity = 66.7%; specificity = 84.8%; *p* < 0.001), followed by MCA PI (AUC = 0.747; *p* = 0.002) and Mod-MPI (AUC = 0.740; *p* = 0.003).

**Figure 3:**
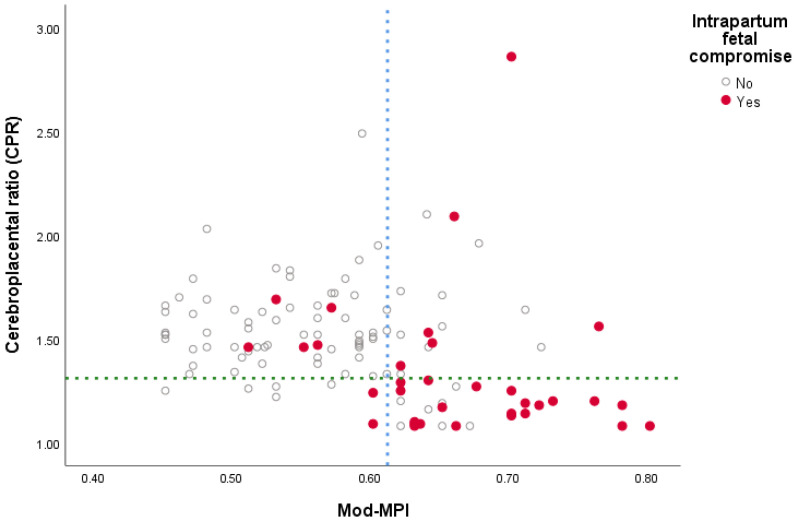
Scatter plot showing the relationship between modified myocardial performance index (Mod-MPI) and cerebroplacental ratio (CPR) stratified by intrapartum fetal compromise (IFC) status. Each dot represents an individual fetus. The blue vertical dashed line indicates the optimal Mod-MPI cut-off value (0.61), and the green horizontal dashed line represents the CPR cut-off value (1.31). Red dots denote pregnancies complicated by IFC, whereas grey dots represent those without IFC.

The relationship between Mod-MPI and CPR stratified by IFC status is illustrated in Figure 3. Cases with and without IFC were classified according to ROC-derived cutoff values (0.61 for Mod-MPI and 1.31 for CPR). A substantial proportion of cases with IFC clustered in the region characterized by elevated Mod-MPI and reduced CPR values. In contrast, the majority of non-IFC cases clustered within the range of low Mod-MPI and normal CPR values. Notably, a subset of cases that developed IFC exhibited elevated Mod-MPI values despite CPR remaining within the normal range.

In univariate logistic regression analysis, AC < 3rd percentile, EFW < 3rd percentile, UA PI > 95th percentile, CPR < 5th percentile, and elevated Mod-MPI (≥0.61) were significantly associated with IFC (Table 4). In multivariate analysis, elevated Mod-MPI (≥0.61) (adjusted OR 5.496; 95% CI: 1.856–16.276; *p* = 0.002) and CPR < 5th percentile (adjusted OR 5.104; 95% CI: 1.699–15.334; *p* = 0.004) remained independently associated with IFC (Table 4). Model fit was supported by the Hosmer–Lemeshow goodness-of-fit test (*p* = 0.349).

## 4. Discussion

### 4.1. Principal Findings

We examined the association between antenatal Doppler and cardiac function parameters and the risk of emergency cesarean delivery due to IFC, as well as CAPO, in term pregnancies complicated by late-onset FGR or SGA. The principal finding was that Mod-MPI values were significantly elevated in IFC cases and demonstrated superior diagnostic performance for predicting IFC compared with conventional Doppler indices. In multivariate regression analysis, after adjustment for birth weight, elevated Mod-MPI (≥0.61) and reduced CPR (<5th percentile) remained independently associated with IFC. With respect to the secondary endpoint, CPR showed higher diagnostic performance than Mod-MPI for predicting CAPO; however, Mod-MPI also exhibited a statistically acceptable level of diagnostic performance.

### 4.2. Interpretation

Significantly elevated Mod-MPI values in late-onset FGR and SGA fetuses support the presence of subclinical cardiac dysfunction. This alteration in cardiac function may represent an early and sensitive adaptive response to placental insufficiency. Such findings are consistent with existing evidence indicating that fetal cardiac dysfunction may precede overt deterioration in conventional arterial Doppler parameters [24,25]. Furthermore, a recent meta-analysis demonstrating significantly higher MPI values in SGA and FGR fetuses compared with healthy controls further corroborates the findings of the present study within a broader evidentiary framework [26].

Placental insufficiency, which underlies FGR, creates a hemodynamic milieu characterized by increased placental vascular resistance and chronic fetal hypoxia [27]. Similarly, SGA fetuses have been shown to exhibit subclinical cardiac dysfunction, suggesting exposure to a comparable degree of hemodynamic stress [25]. In the fetal myocardium, increased afterload and hypoxia-related impairment of intracellular calcium reuptake prolong relaxation phases [28]. Clinically, this manifests as diastolic dysfunction, characterized by shortened ET, a reduced mitral E/A ratio, and prolonged IRT [29,30]. In parallel, increased afterload may also influence systolic dynamics, resulting in prolongation of the ICT. To preserve cardiac output, the fetal heart undergoes adaptive remodeling, transitioning into a more globular configuration [31,32]. Consequently, elevated Mod-MPI emerges as a robust integrative marker that reflects the cumulative diastolic and systolic timing alterations of the fetal heart to increased afterload and hypoxic stress.

Notably, Mod-MPI demonstrated superior predictive performance compared with CPR in identifying cesarean delivery due to acute intrapartum stress. While CPR reflects chronic cerebral redistribution secondary to placental insufficiency, Mod-MPI more directly reflects fetal cardiac reserve [33,34]. The process of labor imposes substantial physiological stress on the fetal circulation as a result of intermittent reductions in uteroplacental perfusion and increased cardiac load associated with uterine contractions [35]. In a multicenter study by Di Mascio et al., CPR was associated with IFC in late-onset FGR, but its diagnostic accuracy was limited [36]. With respect to Mod-MPI, one study reported no significant correlation between this parameter and IFC [37]. Likewise, a recent investigation demonstrated a significant association between low CPR and IFC-related cesarean delivery in SGA fetuses, whereas Mod-MPI did not provide a meaningful contribution [38]. Although these findings appear to contrast with our results, such discrepancies may largely be attributable to limited sample sizes and low event rates in previous studies. In the present study, the inclusion of a more homogeneous FGR/SGA population and a relatively higher number of IFC-related cesarean deliveries enabled a clearer demonstration of Mod-MPI’s ability to reflect cardiac tolerance to acute intrapartum stress. Accordingly, Mod-MPI may offer a theoretical advantage in discriminating fetuses that are prone to decompensation under the acute hemodynamic stress imposed by labor. However, the higher specificity observed for CPR suggests that Mod-MPI may identify a broader group of fetuses at risk, which could potentially increase false-positive identification of fetal compromise if interpreted in isolation.

In this study, both CPR and Mod-MPI demonstrated reasonable discriminatory performance in identifying CAPO as the secondary endpoint. Along similar lines, Jain et al. reported that the combined assessment of Mod-MPI and CPR in the FGR cohort contributed to the prediction of adverse perinatal outcomes [39]. Overall, Mod-MPI appears to reflect clinically meaningful outcomes, including CAPO, beyond its relationship with delivery mode. From a clinical perspective, these findings suggest that antenatal Mod-MPI assessment may offer additional insight into fetal tolerance to intrapartum stress and may assist clinicians in refining risk stratification and delivery planning in pregnancies complicated by late-onset FGR or SGA.

### 4.3. Strengths and Limitations

One of the key strengths of this study is the strict application of contemporary diagnostic criteria. Unlike many previous studies, the exclusive inclusion of term late-onset FGR/SGA pregnancies initially planned for vaginal delivery enhances the applicability of the findings to intrapartum management scenarios where clinical decision-making is uncertain. Another important strength is limiting the interval between ultrasonographic assessment and delivery to 72 h, ensuring close correspondence with intrapartum fetal status. Additionally, the prospective design, clinician blinding, and use of a highly reproducible Mod-MPI technique further strengthen methodological robustness.

However, certain limitations of the present study warrant acknowledgment. Generalization of the findings to broader and more diverse populations may be constrained by the study’s single-center design and relatively modest cohort size. Moreover, the absence of detailed immediate postnatal hemodynamic data (such as the requirement for inotropic support or continuous blood pressure monitoring), as well as long-term neonatal follow-up, precludes more precise evaluation of the relationship between antenatal Mod-MPI values and neonatal cardiovascular adaptation. Detailed information regarding familial predisposition to FGR or SGA was not systematically evaluated, which represents an additional limitation of the present study. Future large-scale, multicenter validation studies are warranted to more clearly elucidate the role of Mod-MPI in assessing intrapartum fetal tolerance and its potential contribution to clinical decision-making.

## 5. Conclusions

In term late-onset FGR or SGA pregnancies scheduled for a trial of labor, Mod-MPI exhibits superior discriminatory performance over standard Doppler parameters in predicting acute hemodynamic stress and IFC risk. Although Mod-MPI does not demonstrate definitive superiority over CPR, its reasonable performance in discriminating CAPO suggests a potential association with clinically meaningful outcomes beyond delivery mode alone. Collectively, Mod-MPI should serve not as a standalone determinant but as a complementary tool for intrapartum risk assessment in selected high-risk late-onset FGR/SGA cases.

## Figures and Tables

**Table 1:** Maternal demographics, clinical characteristics, and perinatal outcomes of the study population stratified by the occurrence of intrapartum fetal compromise.

	Non-IFC (*n* = 86)	IFC (*n* = 34)	*p*
Age (years)	26.5 (20–43)	29.5 (20–39)	0.389
BMI (kg/m^2^)	27.6 (20.2–44.0)	26.1 (22.0–36.8)	0.580
Gravidity	3 (1–7)	3 (1–7)	0.254
Parity	1 (0–5)	1.5 (0–6)	0.158
Smoking status	14 (16.3)	7 (20.6)	0.576
Chronic disease			0.986
Chronic hypertension	9 (10.5)	4 (11.8)	
Pregestational diabetes	2 (2.3)	0 (0)	
Thyroid disease	12 (14.0)	4 (11.8)	
Other	2 (2.3)	0 (0)	
Pregnancy-related complications			0.232
GDM	7 (8.1)	0 (0)	
GIH	17 (19.8)	7 (20.6)	
Intrahepatic cholestasis	4 (4.7)	0 (0)	
Clinical diagnosis			**<0.001**
SGA	67 (77.9)	5 (14.7)	
FGR	19 (22.1)	29 (85.3)	
GA at USG (weeks)	38.0 (36.7–38.4)	37.5 (36.5–38.4)	**<0.001**
AC < 3rd centile	13 (15.1)	17 (50.0)	**<0.001**
EFW < 3rd centile	7 (8.1)	10 (29.4)	**0.003**
Perinatal outcome			
GA at delivery (weeks)	38.1 (37.0–38.4)	37.7 (37.0–38.4)	**<0.001**
Birth weight (g)	2660 (1940–2800)	2467.5 (1885–2785)	**<0.001**
Apgar score < 7 at 5 min	1 (1.2)	2 (5.9)	0.193
NICU admission	4 (4.7)	10 (29.4)	**<0.001**
CAPO	4 (4.7)	11 (32.4)	**<0.001**

Data are presented as median (minimum–maximum) for continuous variables and number (percentage) for categorical variables. Statistically significant *p* values are shown in bold. No cases of perinatal mortality were observed in either group. Abbreviations: IFC, intrapartum fetal compromise; BMI, body mass index; GDM, gestational diabetes mellitus; GIH, gestational induced hypertension; SGA, small for gestational age; FGR, fetal growth restriction; USG, ultrasonography; AC, abdominal circumference; EFW, estimated fetal weight; NICU, neonatal intensive care unit; CAPO, composite adverse perinatal outcome.

**Table 2:** Comparison of fetal Doppler indices and cardiac functional parameters between fetuses with and without intrapartum compromise.

	Non-IFC (*n* = 86)	IFC (*n* = 34)	*p*
UA PI	1.04 (0.69–1.26)	1.10 (0.72–1.44)	**0.001**
Abnormal UA PI (>95th centile)	7 (8.1)	14 (41.2)	**<0.001**
MCA PI	1.55 (1.04–2.15)	1.36 (1.05–2.36)	**0.001**
CPR	1.51 (1.08–2.49)	1.22 (1.08–2.86)	**<0.001**
CPR < 5th centile	9 (10.5)	20 (58.8)	**<0.001**
Ductus venosus PI	0.51 ± 0.10	0.49 ± 0.11	0.421
Mitral E (cm/s)	28.7 (20.1–44.3)	29.4 (20.5–44.2)	0.647
Mitral A (cm/s)	48.7 (35.0–62.6)	49.1 (35.0–62.2)	0.488
Mitral E/A ratio	0.65 ± 0.17	0.62 ± 0.14	0.288
ICT (ms)	41.5 (35–60)	48 (36–59)	**<0.001**
IRT (ms)	52.5 (36–64)	60 (43–64)	**0.004**
ET (ms)	170 (150–188)	158 (150–180)	**<0.001**
Mod-MPI	0.56 ± 0.07	0.65 ± 0.07	**<0.001**

Data are presented as mean ± standard deviation for normally distributed variables, median (minimum–maximum) for non-normally distributed variables, and number (percentage) for categorical variables. Statistically significant *p* values are shown in bold. Abbreviations: IFC, intrapartum fetal compromise; UA, umbilical artery; PI, pulsatility index; MCA, middle cerebral artery; CPR, cerebroplacental ratio; ICT, isovolumetric contraction time; IRT, isovolumetric relaxation time; ET, ejection time; Mod-MPI, modified myocardial performance index.

**Table 3:** ROC analysis of Doppler indices and modified Myocardial Performance Index for predicting intrapartum fetal compromise and composite adverse perinatal outcome.

Variables	Cutoff	AUC	95% CI	Sensitivity (%)	Specificity (%)	*p*
Intrapartum fetal compromise						
UA PI	1.09	0.691	0.571–0.811	55.9	80.2	**0.001**
MCA PI	1.37	0.699	0.594–0.804	55.9	81.4	**0.001**
CPR	1.31	0.759	0.652–0.865	67.6	83.7	**<0.001**
Mod-MPI	0.61	0.835	0.756–0.914	79.4	77.9	**<0.001**
Composite adverse perinatal outcome						
UA PI	1.06	0.668	0.552–0.814	66.7	61.0	**0.036**
MCA PI	1.37	0.747	0.620–0.873	73.3	77.1	**0.002**
CPR	1.24	0.779	0.649–0.908	66.7	84.8	**<0.001**
Mod-MPI	0.59	0.740	0.640–0.840	93.3	60.0	**0.003**

Optimal cut-off values were determined using the Youden index. Bold values indicate statistical significance (*p* < 0.05). Abbreviations: AUC, area under the receiver-operating characteristics curve; CI, confidence interval; UA, umbilical artery; PI, pulsatility index; MCA, middle cerebral artery; CPR, cerebroplacental ratio; Mod-MPI, modified myocardial performance index.

**Table 4:** Univariate and multivariate logistic regression analysis identifying independent predictors of intrapartum fetal compromise.

	Univariate Analysis		Multivariate Analysis	
	Unadjusted OR (95% CI)	*p*	Adjusted OR (95% CI)	*p*
Birth weight (g)	0.996 (0.994–0.998)	**<0.001**		
AC < 3rd centile	5.615 (2.296–13.735)	**<0.001**		
EFW < 3rd centile	4.702 (1.615–13.688)	**0.005**		
Abnormal UA PI (>95th centile)	7.900 (2.816–22.160)	**<0.001**		
CPR < 5th centile	12.222 (4.628–32.279)	**<0.001**	5.104 (1.699–15.334)	**0.004**
High Mod-MPI (≥0.61)	11.221 (4.287–29.368)	**<0.001**	5.496 (1.856–16.276)	**0.002**

The model was adjusted for potential confounders. The optimal cut-off value for the modified myocardial performance index (Mod-MPI) was determined using the Youden index derived from receiver operating characteristic (ROC) curve analysis. Bold values indicate statistical significance (*p* < 0.05). Abbreviations: OR, odds ratio; CI, confidence interval; AC, abdominal circumference; EFW, estimated fetal weight; UA, umbilical artery; PI, pulsatility index; CPR, cerebroplacental ratio; Mod-MPI, modified myocardial performance index.

## Data Availability

The data presented in this study are available on request from the corresponding author due to privacy and ethical restrictions.

## Abbreviations

The following abbreviations are used in this manuscript:

AC	Abdominal Circumference
ALARA	As Low As Reasonably Achievable
AUC	Area Under the Curve
BMI	Body Mass Index
CAPO	Composite Adverse Perinatal Outcomes
CI	Confidence Interval
CPR	Cerebroplacental Ratio
CRL	Crown–Rump Length
CTG	Cardiotocography
DV	Ductus Venosus
DV PI	Ductus Venosus Pulsatility Index
EFW	Estimated Fetal Weight
ET	Ejection Time
FGR	Fetal Growth Restriction
FIGO	International Federation of Gynaecology and Obstetrics
FMF	Fetal Medicine Foundation
ICT	Isovolumetric Contraction Time
IFC	Intrapartum Fetal Compromise
IRT	Isovolumetric Relaxation Time
ISUOG	International Society of Ultrasound in Obstetrics and Gynecology
MCA	Middle Cerebral Artery
MCA PI	Middle Cerebral Artery Pulsatility Index
Mod-MPI	Modified Myocardial Performance Index
NICU	Neonatal Intensive Care Unit
OR	Odds Ratio
PRF	Pulse Repetition Frequency
ROC	Receiver Operating Characteristic
SGA	Small-for-Gestational-Age
STROBE	Strengthening the Reporting of Observational Studies in Epidemiology
UA	Umbilical Artery
UA PI	Umbilical Artery Pulsatility Index
